# Alteration in NMDAR subunits in different brain regions of chronic unpredictable mild stress (CUMS) rat model

**DOI:** 10.1515/tnsci-2022-0255

**Published:** 2022-10-26

**Authors:** Jing Chen, Yanmin Luo, Xin Liang, Xiangru Kong, Qian Xiao, Jing Tang, Yingqiang Qi, Yong Tang, Yun Xiu

**Affiliations:** Molecular Medicine Diagnostic and Testing Center, Institute of Life Science, Chongqing Medical University, Chongqing, 400016, P. R. China; Department of Physiology, Chongqing Medical University, Chongqing, 400016, P. R. China; Department of Pathophysiology, Chongqing Medical University, Chongqing, 400016, P. R. China; Department of Pediatric Surgical Oncology, Children’s Hospital of Chongqing Medical University, Chongqing, 400014, P. R. China; Department of Radioactive Medicine, Chongqing Medical University, Chongqing, 400016, P. R. China; Department of Histology and Embryology, Chongqing Medical University, Chongqing, 400016, P. R. China

**Keywords:** depression, GluN2B, GluN3A, medial prefrontal cortex, corpus callosum

## Abstract

*N*-Methyl-d-aspartate receptor (NMDAR) signaling pathway has been implicated in the pathogenesis and treatment of depression. However, the role of NMDAR subunits in depression is still unclear. In this study, alteration in all seven NMDAR subunits in several brain areas of rats exposed to chronic unpredictable mild stress (CUMS), an animal model of depression, was detected. Our findings demonstrated that: (1) CUMS could induce a reduction in sucrose preference, an indicator of typical depression-like behaviors; (2) CUMS significantly reduced the NMDAR subunits of GluN2B and GluN3 in the medial prefrontal cortex (mPFC), but not altered all seven NMDAR subunits in hippocampus and corpus callosum of rats; (3) subunit composition of NMDARs in corpus callosum was different from that in mPFC, PFC and hippocampus; and (4) the mRNA expressions of GluN2B, GluN3A and GluN3B in mPFC as well as mRNA expression of GluN2C in corpus callosum were correlated to sucrose preference in rats. These findings suggested that GluN2B and GluN3 in mPFC may contribute to the pathophysiology of depression.

## 1 Introduction

Depression is a common illness characterized by significant and persisting loss of pleasure or interest in usual activities that severely limits psychosocial functioning and diminishes the quality of life [1]. In western countries, mere major depressive disorder (MDD) affects approximately 17% of the population, which results in enormous personal and economic burden and is on pace to be the leading cause of disability worldwide [2]. However, current pharmacological treatment for depression is not very effective and immediately. Take monoamine reuptake blockers as examples, fluoxetine, sertraline or citalopram, often requires weeks to months to yield a therapeutic response. Furthermore, approximately one-third of patients are resistant to the multiple prescriptions and/or these drug combinations [3]. Therefore, there is a clear need for new effective rapid-acting antidepressant medications.

It is remarkably encouraging for investigators when they found that ketamine, an *N*-methyl-d-aspartate receptor (NMDAR) antagonist developed as an anesthetic drug, produces rapid antidepressant actions [4]. It is reported that even a single low dose of ketamine can produce a rapid antidepressant effect within hours, those effects can last for 3–7 days [5,6,7]. In March 2019, a nasal form of ketamine, esketamine, has received U.S. Food and Drug Administration approval for treatment-resistant depression [8]. However, there are several questions unresolved in ketamine’s and its analogs’ antidepressant action. For instance, the questions why ketamine has distinct effects on electro-physiologies and behaviors in depressed and healthy subjects [9] and what neurobiological mechanisms underlie the rapid and sustained antidepressant actions of ketamine [2] were proposed. Given that ketamine is an NMDAR antagonist, one of the new strategies for antidepressant drug development focuses on blocking the function of NMDARs. Due to the indispensable role of NMDARs in synaptic development, learning memory and cognition, there is a growing interest in exploring the pharmacological heterogeneity of NMDARs with different subunits and its role in depression.

NMDARs are tetrameric ionotropic glutamate receptors, composed of two GluN1 subunits with two GluN2 or GluN3 subunits [10,11]. Subunits GluN1, GluN2A, GluN2B, GluN2C and GluN2D in depression were investigated in previous studies. For instance, the team lead by Duman found no significant difference of GluN1, GluN2A, GluN2B, GluN2C and GluN2D mRNA expression in dentate gyrus (DG) and CA1 subfield of hippocampus between subjects diagnosed with MDD compared to healthy subjects matched for sex and ages [12], while other researchers found the reduced levels of GluN2A and GluN2B subunits in the anterior prefrontal cortex (PFC) [13] and the elevated levels of the GluN2C subunit in the locus coeruleus [14] in depression subjects. Moreover, it was proved that both selective GluN2B antagonist (Ro 25-6981) and GluN2A receptor antagonist (NVP-AAM077) have potential antidepressant activity, such as rapidly ameliorating chronic unpredictable mild stress (CUMS)-induced anhedonia and anxiogenic behaviors [15,16,17,18]. However, the GluN3 subunit in depression was less studied. Hence, the first aim of this study was to investigate the alteration in GluN3 subunit in animal model of depression. Besides, the changes in GluN1 and GluN2 in depression found in previous studies are controversial [12,13,14]. These controversies are partly stemmed from the fact that different region of brain was investigated. Thus, the second aim of this study was to investigate changes in NMDAR subunits in different brain regions, including hippocampus, PFC, corpus callosum and the specific subfield of PFC, medial prefrontal cortex (mPFC). Collectively, the present study was designed to find the “possible subunit” of NMDARs involved in depression-like behavior induced by CUMS.

## 2 Materials and methods

### 2.1 Animals

Male Sprague-Dawley rats (150–200 g) were obtained from Animal Experimental Center of Chongqing Medical University. Animals were maintained under standard laboratory conditions and housed 4–5 per cage (29 cm × 22 cm × 14 cm) with sufficient food and water. After 1 week of adaption to surrounding environment, rats were randomly divided into control group (*n* = 23) and CUMS group (*n* = 26). The control rats were housed 4–5 per cage as the same as before, and the CUMS rats were housed one per individual cage.


**Ethical approval:** The research related to animals’ use has been complied with all the relevant national regulations and institutional policies for the care and use of animals. All experiments were in accordance with the guidelines for the protection and use of laboratory animals. Procedures were followed the National Institutes of Health Guide for the Care and Use of Laboratory Animals.

### 2.2 CUMS protocol

Rats in the CUMS group were exposed to a sequence of unpredictable stresses, while rats in the control group were reared under a standard laboratory condition without any stress stimuli. The chronic unpredictable mild stressors, including water/food deprivation, cage tilting, damp bedding, intermittent illumination, strobe light, tail clip, empty bottle, cold/hot stress, restraint, tail pinch, foot electric shock and noise, were designed to prevent adaption (see Table 1). The CUMS lasted for 8 weeks, and each stimulus was presented no more than twice a week [19].

**Table 1:** Protocol of chronic unpredictable mild stress

Days	Stressors
D1	Wet bedding overnight; cage tilt 45° overnight
D2	24 h water deprivation; light on 24 h
D3	electric shock; night strobe
D4	Immobilization; light off 24 h; empty bottle stimulation
D5	Noisy; vibration; cold environment at 4°C
D6	Food deprivation; reversal of the light/dark cycle (light off 12 h and light on overnight)
D7	SPT

### 2.3 Behavioral tests

The sucrose preference test (SPT) was conducted to verify depression-like behaviors before and after the CUMS intervention [19,20,21,22]. Before the test session, rats were deprived of water for 24 h, and then each rat in an individual cage was exposed to two bottles, one of which was filled with a 1% sucrose solution and the other of which was filled with fresh water. The two bottles were exchanged the position after 12 h. The sucrose solution consumption and water consumption in 24 h were measured respectively. The percentage of sucrose consumption to total liquid consumption reflects the sucrose preference.

### 2.4 Tissue preparation

Tissues for real-time quantitative polymerase chain reaction (qPCR) and western blotting were prepared by method as follows. After behavioral tests, five rats from each group were deeply anesthetized with 1% pentobarbital sodium injected intraperitoneally. The brains were quickly dissected and split into two hemispheres on ice. One cerebral hemisphere was separated to obtain the tissue of mPFC, remaining PFC in which the mPFC was excluded (PFC), corpus callosum and hippocampus for qPCR. The tissues of mPFC, PFC corpus callsoum and hippocampus from the other hemisphere were prepared for western blotting.

### 2.5 Real-time qPCR

Total RNAs from tissues were extracted with TRIzol reagent according to the manufacturer’s manuals. The total RNA was first reverse transcribed to cDNA using PrimerScript™ RT reagent Kit with gDNA Eraser (Takara RR047A). SYBR Green PCR mix (Takara RR820A) was used for fluorescence PCR, in 10 μL of reaction mixture containing 4 μL cDNA and 1 μL (1 pmol/μL) primers with a protocol of 95°C for 2 min, then 95°C for 10 s and 56°C for 30 s. Protocols of step 2 and step 3 were repeated for 39 cycles. For RNA internal control, housekeeping gene *ACTIN* was used to normalize the mRNA expression of NMDAR subunits. The primers of all seven NMDAR subunits and *ACTIN* gene are shown in Table 2. Results of the qPCR were represented as Ct values, and comparative ^ΔΔ^CT methods were used to calculate the relative mRNA expression of NMDAR subunits.

**Table 2:** The primers used in the current study

Gene name	Primers sequences	Application
*Grin1*	Fwd: 5′-TCCTACACAGCTGGCTTCTACAG-3′	Quantitation for rats
	Rev: 5′-CACCGTGCGAAGGAAACTCA-3′	
*Grin2a*	Fwd: 5′-CCCAAAGAGTTTCCATCAGGT-3′	
	Rev: 5′-CGGCAGTGGTTAAGATCCCA-3′	
*Grin2b*	Fwd: 5′-GGGTCACGCAAAACCCTTTC-3′	
	Rev: 5′-CCTTGTTTTTGACGCCCCTG-3′	
*Grin2c*	Fwd: 5′-GGTATCAAGGAGCAACGGCA-3′	
	Rev: 5′-CCCTTGGTGAGGTTCTGGTT-3′	
*Grin2d*	Fwd: 5′-AGGAAGGCTGGGACCTAAGA-3′	
	Rev: 5′-TTCCTCCAAACCAGGGGTTC-3′	
*Grin3a*	Fwd: 5′-ATAGTGCGCCACGAGTTTCC-3′	
	Rev: 5′-CTTCCTGGCAGAGCAACAAG-3′	
*Grin3b*	Fwd: 5′-CCCTGTACGAATGGCGAAGT-3′	
	Rev: 5′-GTGCGTCCAAAGAGAATGGC-3′	
*Actin*	Fwd: 5′-AACCGTGAAAAGATGACCCAG-3′	
	Rev: 5′-AGGCATACAGGGACAACACA-3′	

### 2.6 Western blotting

Tissues of specific brain region were cut into small pieces and lysed in phenylmethylsulfonyl fluoride-containing protein lysis buffer. After centrifuging with a speed of 12,000 rpm at 4°C for 10 min, the lysates were collected. Then, protein concentration of the sample was determined by the copper-based total protein quantification method. After this process, the lysates were boiled with loading buffer for 10 min and stored at −20°C. When sodium dodecyl sulfate-polyacrylamide gel electrophoresis was conducted, the concentration of protein samples was adjusted to ensure the equivalent amount of loading protein. Then, the proteins were separated by vertical electrophoresis for 90 min and transferred to the polyvinylidene difluoride (PVDF) membrane. This membrane was blocked with 5% bovine serum albumin at room temperature for 2 h. Next, the PVDF membrane was incubated with the primary antibodies, anti-GluN2B antibody (Abcam ab65783 1:1,000), anti-GluN3A (Biorbyt orb 157999 or a gift form Prof. Watanabe) and anti-β-actin antibody (Bioss bs-0061R 1:5,000) at 4°C overnight. After that, the secondary antibody, goat anti-rabbit IgG (1:10,000), was added for incubation at 37°C for 1 h. At last, the immunoreactive protein bands were visualized with an ECL luminescent detection kit. The target protein was quantitatively analyzed by the ImageJ software. Expression of target protein was normalized to β-actin before comparation between different groups.

### 2.7 Statistical analysis

All statistical analyses were performed using SPSS 25.0 software. The Shapiro–Wilk test was used to evaluate whether the data were normally distributed. If data were normally distributed and have similar variance, two tail unpaired Student’s *t*-test was used; otherwise, Mann–Whitney *U* test was used. The data of body weight were analyzed using repeated measures analysis of variance (ANOVA). Pearson method was used in correlation analysis between NMDAR subunit mRNA level and sucrose preference. *p* < 0.05 is considered statistically significant.

## 3 Results

### 3.1 Rats in the CUMS group exhibited depression-like symptom

**Figure 1:**
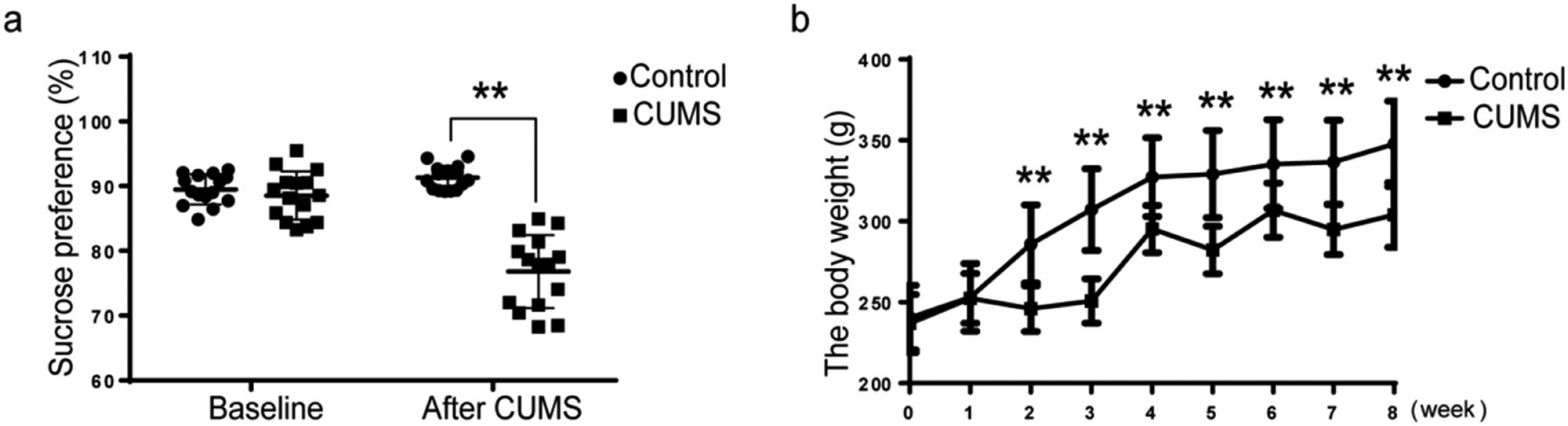
CUMS induces depression-like behavior in rats. (a) Sucrose preference in CUMS rats is significantly decreased compared to that in control rats. (b) From the third week of CUMS exposure, the body weight of the rats in the CUMS group is significantly lower than that of rats in control group. The data are expressed as the mean ± SD (*n* = 23 in the control group and *n* = 26 in the CUMS group). **p < 0.01.

CUMS is the most commonly used, reliable and effective strategy to make rodent model of depression. In our current study, before the CUMS intervention, there was no significant difference in the sucrose preference between the control group and the CUMS group (*p* = 0.167). At the end of the CUMS intervention, the sucrose preference percentage of the rats in the CUMS group was significantly lower than that in the control group (*p* = 1.11 × 10^−7^ < 0.01; Figure 1a). This result indicated that rats in CUMS group exhibited depression-like symptom.

Besides, the change in body weight in rats exposed to CUMS was consistent with previous studies [19,20]. Before the CUMS, the body weights of rats in the CUMS group were comparable to those of rats in the control group (*p* = 0.431). Two weeks after stresses, the weight gains of rats in the CUMS model group were lower than those of rats in the control group, and the body weights significantly differed between the control group and the CUMS group from the third week to the eighth week (repeated measures ANOVA *F*_(1,47)_ = 37.344, *p* = 1.828 × 10^−7^ < 0.01; Figure 1b).

### 3.2 CUMS induced a decrease in expression of GluN2B, GluN3A and GluN3B in mPFC, but not in PFC, hippocampus and corpus callosum

**Figure 2:**
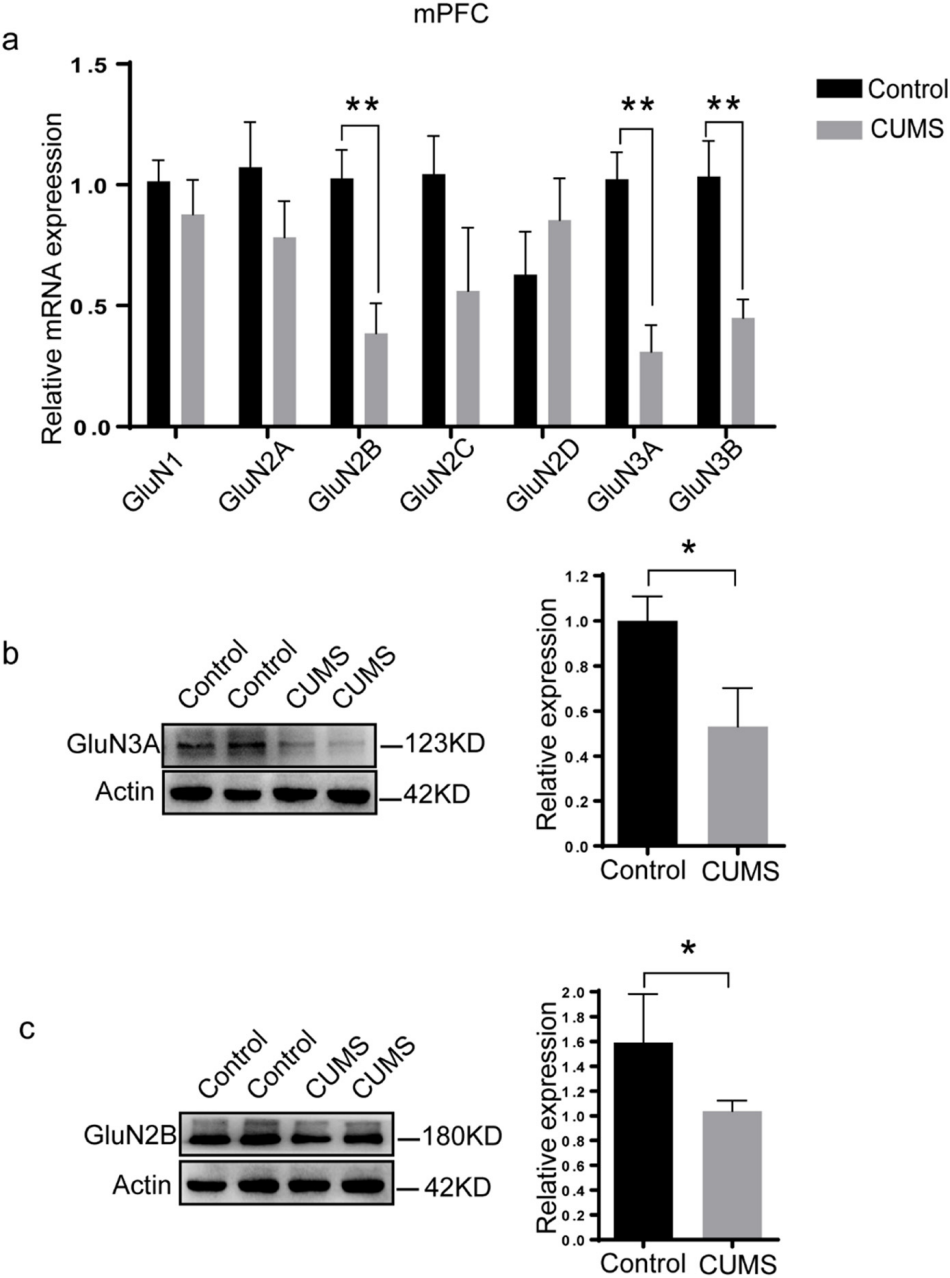
CUMS induces a reduction in expressions of GluN2B, GluN3A and GluN3B in the mPFC. (a) The mRNA expression of seven NMDAR subunits in the mPFC. (b) Western blotting results of GluN3A in the mPFC. (c) Western blotting results of GluN2B in the mPFC. The data are expressed as the mean ± SD (*n* = 5 per group). *p < 0.05, **p < 0.01.

**Figure 3:**
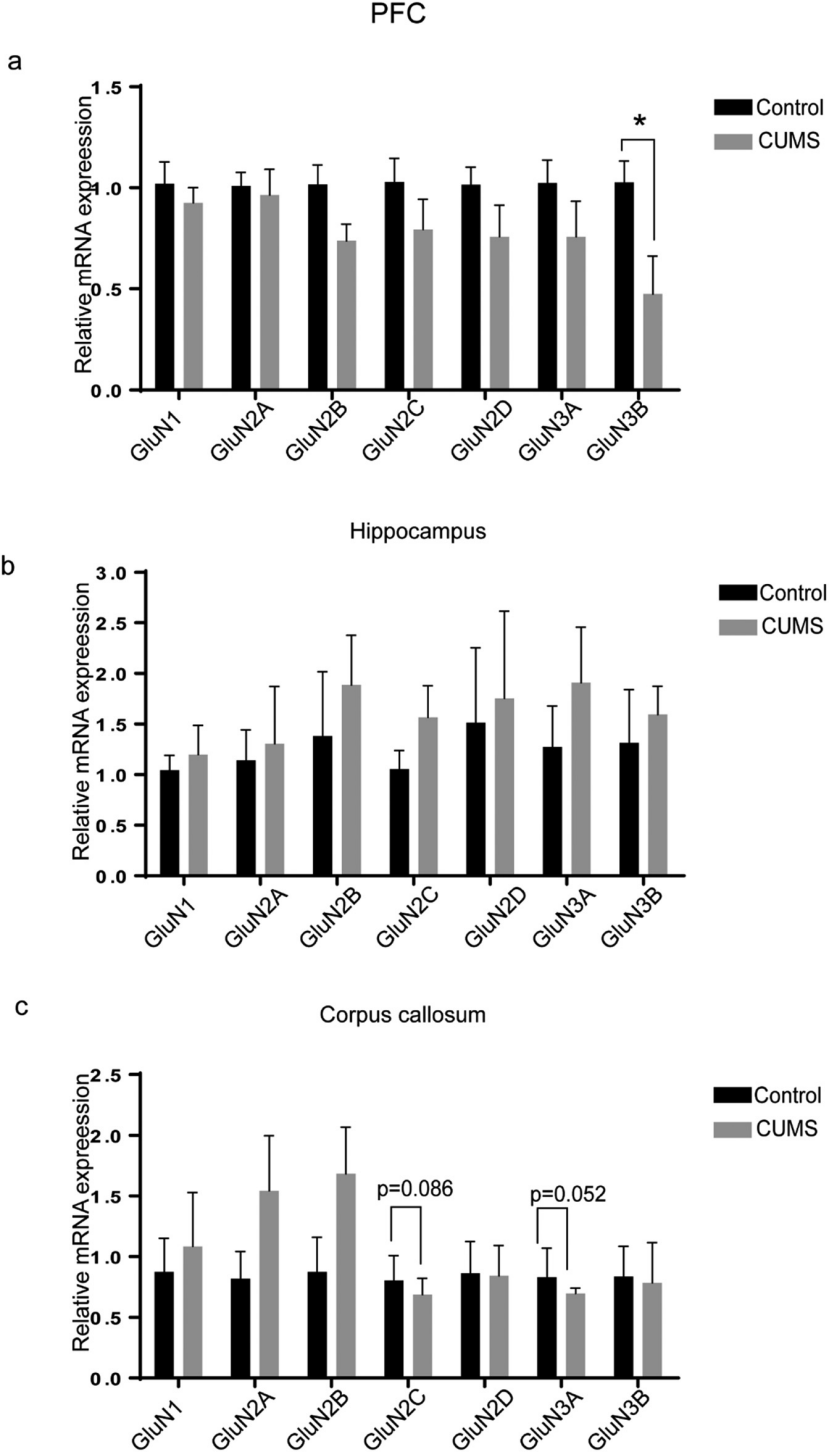
The mRNA expressions of seven NMDAR subunits in the PFC, hippocampus and corpus callosum after CUMS exposure. (a) The mRNA expression of seven NMDAR subunits in the PFC where the mPFC is excluded. (b) The mRNA expression of seven NMDAR subunits in the hippocampus. (c) The mRNA expression of seven NMDAR subunits in the corpus callosum. The data are expressed as the mean ± SD (*n* = 5 per group). *p < 0.05.

mPFC and hippocampus have been deemed to be important brain regions vulnerable to stress exposure and associate with depression [23,24]. In the present study, we found that the mRNA expression of GluN2B (*p* = 0.006 < 0.01), GluN3A (*p* = 0.002 < 0.01) and GluN3B (*p* = 0.008 < 0.01) in mPFC was significantly downregulated in CUMS rats compared to those in control rats (Figure 2a). The expression of GluN3A protein (*p* = 0.016 < 0.05; Figure 2b) and GluN2B protein (*p* = 0.015 < 0.05; Figure 2c) in the mPFC of CUMS rats was significantly lower than that of control rats. However, in the other parts of PFC where mPFC was removed, only GluN3B mRNA expression was significantly decreased (*p* = 0.034 < 0.05, Figure 3a) compared to that in control rats. There was no significant difference in all seven NMDAR subunits in the hippocampus between control rats and CUMS rats (Figure 3b). Given accumulating evidence supports that impairment of white matter plays a key role in the pathogenesis of depressive disorders [25,26,27,28,29,30], we also investigated the alterations in all seven NMDAR subunit mRNA in the corpus callosum of CUMS rats in the current study. Although no significant alterations in NMDAR subunit mRNA were found in the corpus callosum of CUMS rats, it must be underlined that there is a trend of decrease in the mRNA level of GluN2C (*p* = 0.086) and GluN3A (*p* = 0.052) in the corpus callosum of CUMS rats (Figure 3c).

### 3.3 Subunit composition of NMDARs in corpus callosum was different from that in PFC, mPFC and hippocampus

**Figure 4:**
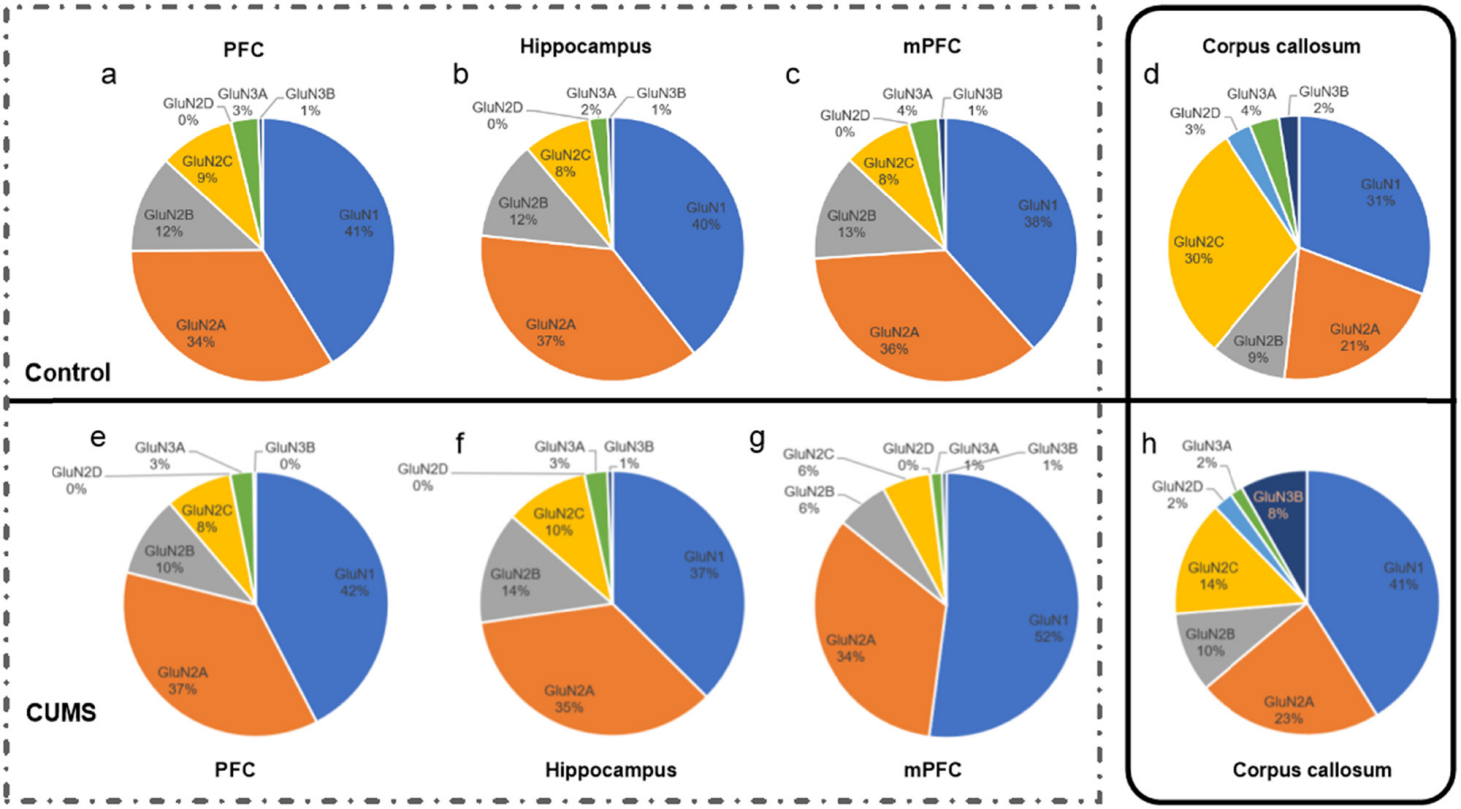
Subunit composition of NMDARs in different brain regions. Subunit composition of NMDARs in the PFC, hippocampus, mPFC and corpus callosum of rats in the control group (a–d) and in the CUMS group (e–h) according to the relative mRNA expression of GluN1 subunit within its own region. Subunit composition of NMDARs in the corpus callosum with the high proportion of GluN1, GluN2C-D and GluN3A-D (solid box) is different from that in PFC, hippocampus and mPFC with the high proportion of GluN1 and GluN2A-B (dashed box).

NMDARs are widely expressed throughout the central nervous system, but their number, localization and subunit composition are strictly regulated and differ in a cell- and synapse-specific manner [11]. To assess the difference in subunit composition of NMDARs in different brain regions, proportions of NMDAR subunits were estimated according to the GluN1 mRNA level within its own region. The data showed that NMDARs in the PFC were mainly composed of GluN1 (41%), GluN2A (34%), GluN2B (12%), GluN2C (9%), GluN3A (3%) and minute quantity of GluN2D (0.07%) and GluN3B (0.53%). The subunit composition of NMDARs in the mPFC and in the hippocampus was similar to that in the PFC (Figure 4a–c, dashed box). However, subunit composition of NMDARs in the corpus callosum was different from that in the above-mentioned gray matter regions, with lower GluN1(31%), GluN2A (21%) and GluN2B (9%), but higher GluN2C (30%), GluN3A (4%), GluN2D (3%) and GluN3B (2%) (Figure 4d, solid box). The changes in NMDAR subunits in mPFC, PFC, hippocampus and corpus callosum after CUMS are shown in Figure 4e–h.

### 3.4 The mRNA levels of GluN2B, GluN3A and GluN3B in mPFC and GluN2C in corpus callosum were correlated to sucrose preference

**Figure 5:**
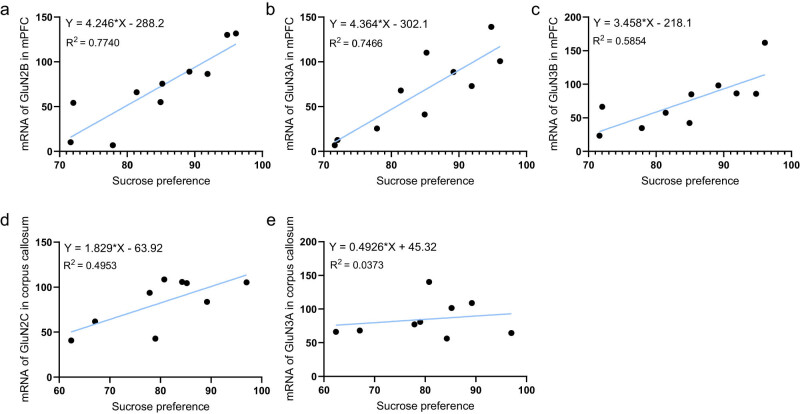
Correlation between NMDAR subunit and sucrose preference. (a–c) The mRNA levels of GluN2B, GluN3A and GluN3B in mPFC are correlated with sucrose preference score in rats. (d and e) The mRNA level of GluN2C rather than GluN3A in corpus callosum is correlated with sucrose preference score in rats.

We next analyzed the correlation of NMDAR subunit and the sucrose preference (Figure 5). The results showed that the mRNA levels of GluN2B (*r* = 0.880; *p* = 0.001) and GluN3A (*r* = 0.864; *p* = 0.001) in the mPFC were highly correlated with the sucrose preference scores in rats. The mRNA levels of GluN3B in the mPFC (*r* = 0.765; *p* = 0.010) and GluN2C in the corpus callosum (*r* = 0.704; *p* = 0.034) were also correlated with the sucrose preference scores in rats. However, the mRNA level of GluN3A (*r* = 0.193; *p* = 0.619) in the corpus callosum was not correlated with the sucrose preference scores in rats.

## 4 Discussion

Findings of ketamine’s effective and fast-acting antidepressant effect indicate the NMDAR system involvement in depression [4,5,6,7]. Further scientific findings indicate that the NMDAR subunit-selective compounds may be novel therapeutically agents for depression [15,16,17,18]. However, there are only a small number of previous studies focusing on the NMDAR subunits in depression, and the results are incomplete and controversial. In this study, all seven NMDAR subunits (including the GluN3 subunit, which used to be less investigated in previous studies) in specific brain regions of CUMS rats (including mPFC, PFC, hippocampus, and corpus callosum) are investigated. The “possible NMDAR subunit(s)” involved in the pathophysiology of depression were expected to be found through our current study.

A large number of previous studies have proved that GluN2B-containing NMDARs play an important role in depression. For example, some researchers found the downregulation of GluN2B in the PFC [13] or the perirhinal cortex [31] of patients with MDD. Recently, low hippocampus exNMDAR function was demonstrated in the mice susceptible to the chronic social defeat stress, and raising exNMDAR function could prevent the social avoidance behaviors in stressed mice [32]. More importantly, extrasynaptic NMDARs or nonsynaptic NMDARs are the GluN2B-containing NMDARs [33]. Our finding provided another proof that there is a reduction in GluN2B-containing NMDARs in CUMS rat, an animal model of depression. Importantly, there was a strong positive correlation between the mRNA level of GluN2B in mPFC and sucrose preference score in rats, which indicated that GluN2B in mPFC might be involved in depression. Nevertheless, some researchers reported that CUMS can lead to the upregulation of phosphorylated GluN2B protein in the mPFC of rats [34]. And selective GluN2B antagonism or ketamine could rapidly ameliorates CUMS-induced anhedonia behaviors in an mTOR-dependent manner [16]. It is difficult to interpret the contractionary findings due to the “double-edge sword” effect of GluN2B [35]. On the one hand, forebrain overexpression of GluN2B could alter synaptic plasticity and enable the mice with GluN2B overexpression to exhibit “smarter” performance in cognitive tasks compared to their wild-type counterparts [36]; on the other hand, genetic deletion of GluN2B in cortical neurons enables the knockout mice to be less susceptible to stress-associated changes in depression-like behavior [18]. Those controversial findings suggest that further studies on the role of GluN2B in depression are in need.

Besides the GluN2B, GluN3A and GluN3B subunits were also found deceased in the mPFC of CUMS rats in our current study. Although many studies have tested the expression of GluN1, GluN2 (A–D) in MDD patients or rodent model of depression [15,16,17,18,37], few studies have investigated GluN3 subunits. The only one study focusing GluN3A was conducted by Muller and Meador-Woodruff, in which they investigated the GluN3A mRNA expression in subjects with schizophrenia, major depression or bipolar disorder [38]. Nevertheless, they found no alteration in GluN3A mRNA expression in the dorsal lateral prefrontal cortex (DLPFC) or the inferior temporal neocortex of MDD. The different brain regions or whether receiving drug treatment may contribute to the different results. As the authors emphasized in their article that GluN3A alterations in schizophrenia and bipolar disorder existed only in the DLPFC but not in inferior temporal cortical regions, it is likely that different brain regions exhibit distinct neurochemical abnormalities [38]. Moreover, only GluN3A mRNA was investigated in previous research while both mRNA and protein of GluN3A were detected in our current study. To our knowledge, it is the first time to provide the direct evidence of GluN3A downregulation in a rat model of depression. The strong correlation between GluN3A mRNA level in mPFC and sucrose preference suggested that GluN3A may be another subunit involved in depression-like behavior induced by CUMS.

mPFC and hippocampus have been deemed to be important brain regions vulnerable to stress exposure and associate with MDD in previous studies [23,24]. In the present study, the GluN2B, GluN3A and GluN3B subunits in mPFC as well as GluN3B subunit in PFC were found decreased in CUMS rats compared to control rats. However, no changes in GluN1, GluN2A, GluN2B, GluN2C and GluN2D subunits were found in the hippocampus of CUMS rats, which is in line with previous finding in hippocampal sub-regions (DG or CA1) of subjects with MDD [12]. This suggested that PFC, especially mPFC, was the specific region in which NMDAR system plays an indispensable role in depression while the NMDAR system in the hippocampus may not participate in CUMS-induced depressive behaviors. Furthermore, the distinct changes in NMDAR subunits found in different brain regions, including PFC, mPFC, hippocampus and corpus callosum, also suggested that specific brain region should be taken into consideration when a new biomarker was expected to be found in some neuropsychiatric disorders.

Last but not least, NMDA receptors were previously reported to express on oligodendrocyte processes [39,40] and seemed to be a promising target for treating white matter damage in acute and chronic diseases [41]. In addition, numerous studies have shown that depression or chronic social stress was associated with white matter damages [25,26,27,28,29,30]. Therefore, NMDAR subunits in the corpus callosum of rats exposed to CUMS were investigated in the current study. Although no significant alterations in all seven NMDAR subunits were found in the corpus callosum of CUMS rats, the trend to decrease in GluN2C and GluN3A mRNA in the CUMS group must be underlined. In addition, we found that the subunit composition of NMDARs in the corpus callosum differed from the constitution of NMDARs in the PFC, demonstrated by abundant GluN2C subunits in the NMDARs in the corpus callosum. The subunit composition of NMDARs in the PFC was similar to that in the mPFC and in the hippocampus. Those different constitutions of NMDARs would result in quite different functions and thus leading to distinct signal pathways in CUMS rats. Interestingly, it has been reported that NMDARs on oligodendrocytes were mainly composed with GluN2C and GluN3A subunits [39,41,42,43]. Moreover, Pearson analysis showed that GluN2C mRNA expression in corpus callosum was correlated to sucrose preference. Our results underlined the importance of future studies with larger sample size, focusing on the role of GluN2C and GluN3A in white matter damages as well as in depression. However, there are some limitations in our experiment. One of these limitations is that proportions of NMDAR subunits in different brain regions were not statistically analyzed. It is because that samples from different brain regions were not in the same qPCR test, thereby with different amplification efficiency. Eventually, the composition of NMDAR subunit was only calculated based on the GluN1 expression within its region. The other limitation is that only qPCR was used to detect the subunit composition of NMDARs. Diverse detecting methods, such as western blotting, immunohistochemical technique or pharmacological molecular manipulation, should be used in future studies.

## 5 Conclusion

In conclusion, we systematically examined all seven NMDAR subunits in different brain regions of CUMS rats and found that “the possible NMDAR subunits” involved in depression-like behaviors may be the GluN2B, GluN3A and GluN3B in mPFC. In addition, the different changes in subunit composition of NMDARs in different brain regions emphasized that specific brain region should be taken into consideration when a new biomarker was expected to be found in some neuropsychiatric disorders.
